# Bilateral Slipped Capital Femoral Epiphysis Fixation Failure Including the Prophylactic Side: A Case Report

**DOI:** 10.7759/cureus.23291

**Published:** 2022-03-18

**Authors:** Canon C Cornelius, Surya Mundluru

**Affiliations:** 1 Orthopedics, University of Texas Health Science Center at Houston, Houston, USA

**Keywords:** pediatric orthopedics, vitamin d deficiency, pediatrics, fixation failure, slipped femoral epiphysis

## Abstract

Slipped capital femoral epiphysis (SCFE) is a common problem treated by pediatric orthopedic surgeons. A 13-year-old male presented with right-sided hip pain. Both sides, symptomatic and asymptomatic, were treated with a single 7.3-mm screw. The patient returned with symptoms to the bilateral hips 16 months after the procedure. He was treated with removal of hardware and revision fixation with a good outcome. We report a rare case of fixation failure in bilateral SCFE with an excellent outcome. We highlight the importance of quick recognition of failure before displacement and a strategy for hardware removal.

## Introduction

Slipped capital femoral epiphysis (SCFE) affects on average nearly 11 out of 100,000 children with a male predominance [1]. Although multiple treatments for SCFE have been described, a single centrally placed cannulated screw is the most commonly utilized treatment. Even though there are biomechanical advantages to using two screws, this technique is reserved for unstable slips [2]. Bilateral pain, young age, and possible follow-up concerns can lead to the recommendation for contralateral prophylactic pinning [3,4]. While the complications of avascular necrosis, progression, femoral head deformity, and chondrolysis are commonly considered, a rare complication is cannulated screw failure [5,6]. Here, we report a case of bilateral cannulated screw failure despite one side having only minimal displacement and the other side being prophylactically protected due to the patient’s skeletal immaturity. We describe how the broken screws were removed and our revision fixation. To our knowledge, this is the first case of bilateral screw failure including a prophylactically pinned hip to date.

## Case presentation

**Figure 1:**
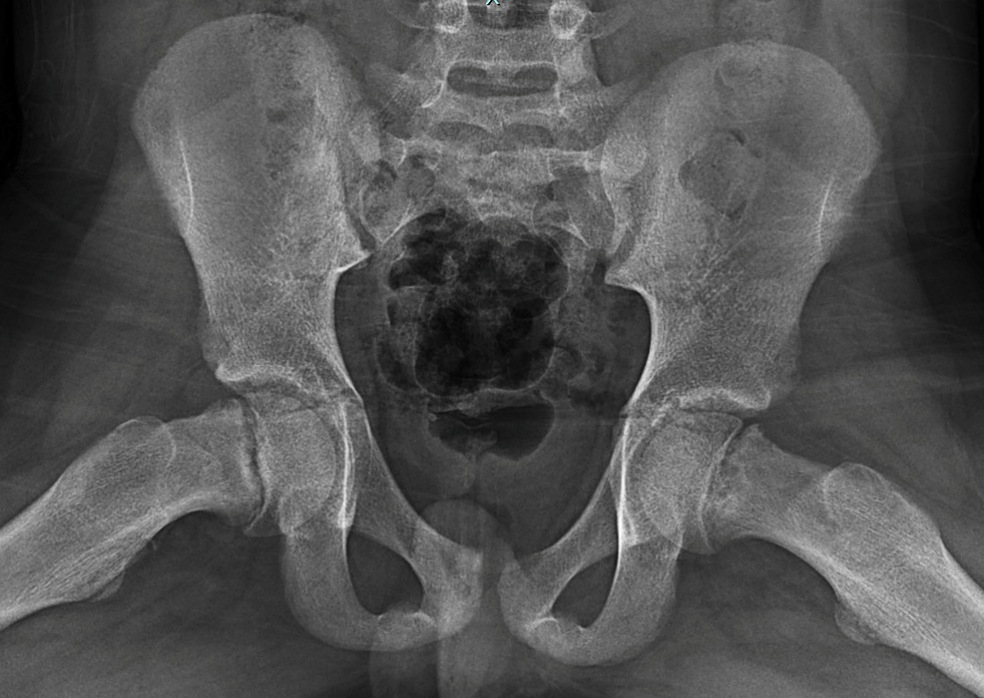
Frog bilateral hips showing right-sided physeal widening.

A 13-year-old male presented to our outpatient pediatric orthopedic clinic with a one to two-week history of right-sided progressive hip pain with ambulation. According to reports, the patient probably had a fall a few weeks prior but the pain was possibly noted for some time before. On physical examination, the patient was noted to have external rotation to 60 degrees on a bilateral hip examination. He had -5 degrees of internal rotation bilaterally. He had pain in his groin on the right side greater than the left. He was able to ambulate but had a mildly antalgic gait on the right side. He had referred knee pain on the right side as well. Anteroposterior (AP) pelvis and frog-leg bilateral hips were performed. The frog leg is shown in Figure 1.

Radiographs were notable for right-sided physeal widening most notable on the frog lateral view, and slight inferior displacement of the epiphysis relative to the metaphysis on the AP. The left hip was otherwise unremarkable. Labs were drawn at this appointment that showed a low vitamin D level, but a normal thyroid-stimulating hormone. The patient had a bone age hand assessment of 13 years of age. He was provided crutches and a wheelchair to reduce weight-bearing. He was scheduled for surgery for in situ screw fixation of the right proximal femur and prophylactic fixation of the left proximal femur. Radiographs were taken in the hospital which showed no changes from prior.

**Figure 2:**
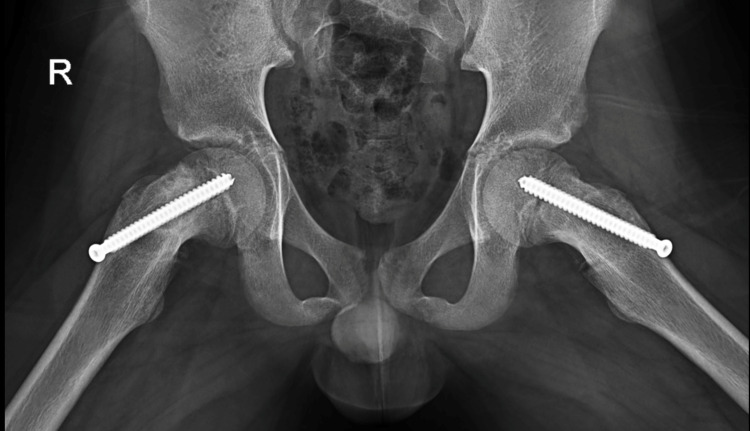
Nine-month follow-up frog bilateral hip X-rays.

He was taken to the operating room where two 7.3 mm × 85 mm fully threaded cannulated screws were placed transphyseal into the central portion of bilateral femoral heads with no complications. No formal capsular decompression was performed. He was discharged with no complications on postoperative day one with notable improvement in his hip pain on range of motion assessment. The patient was seen as an outpatient twice once at two weeks and then at nine months with no complaints of pain and radiographs that appeared unchanged, as shown in Figure 2.

**Figure 3:**
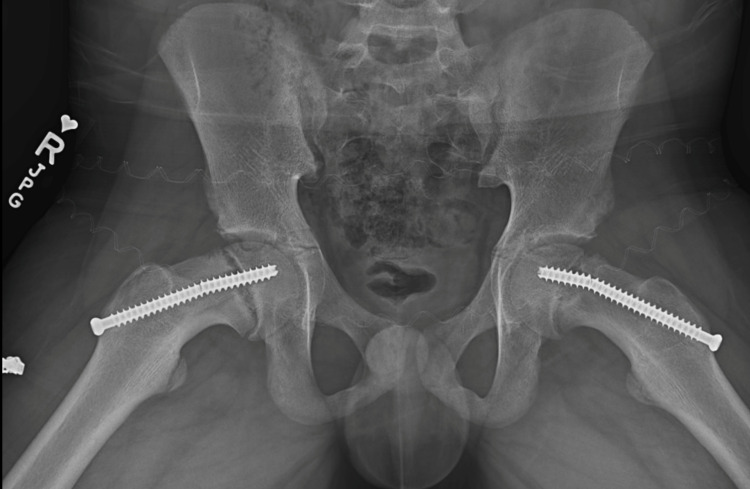
Frog bilateral hips showing bilateral screw failure and progressive slip of the right and left sides.

He was lost to follow-up till 16 months postoperative when the patient’s family called the office stating that his pain had returned. His body mass index increased from 19.5 to 23.5 kg/m^2^ during this time frame. It was described as being milder compared to the initial presentation but present. This was not precipitated by any traumatic event. His physical examination was mostly benign with a normal range of motion and minimal to no pain on walking. Radiographs taken at this time revealed bilateral screw failure with the progression of displacement (Figure 3).

**Figure 4:**
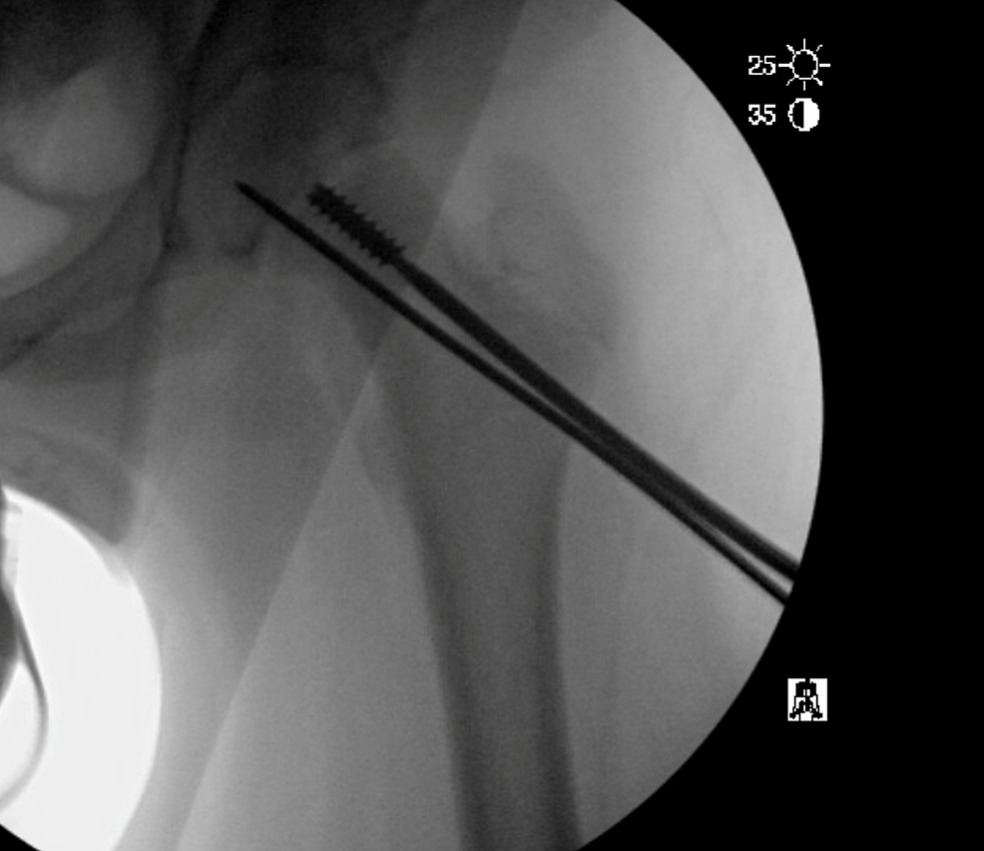
Conical extractor being used to remove the retained broken screw.

He was scheduled for removal of the fractured screws and revision procedure. We began by prophylactically pinning the epiphysis to prevent further displacement. The screw was then recannulated with a wire to allow for easy placement of the cannulated screwdriver. The near side of the screw was removed but the far end remained. At this point, the Conical Extractor (Synthes, Raynham, MA, USA) was used to remove the retained distal portion of the screws bilaterally (Figure 4).

Three Synthes cannulated screws were placed bilaterally on the right, all three were 6.5-mm fully threaded screws in new screws tracts, and on the left, there were two 6.5-mm fully threaded and one 7.3 mm screw. The 7.3-mm screw was placed in the same start point as the prior screw due to the lack of available space for a new screw trajectory in that hip.

Postoperatively, he was seen by the pediatric endocrinology team which ordered a more extensive endocrine workup which showed a continued vitamin D deficiency despite supplementation. They also recommended an outpatient workup with a dual-energy X-ray absorptiometry scan and the possibility of a pediatric genetic consult. He was cleared for discharge by physical therapy, orthopedics, and endocrinology on postoperative day two with no complications.

The patient missed his initial postoperative appointment but presented at the three-month follow-up. According to the family report, the patient was compliant with non-weight-bearing for the first six weeks but progressively began to weight bear thereafter. His pain had completely improved with full painless flexion of bilateral hips, internal rotation of 5 degrees, and a painless gait. His radiographs showed intact hardware with no signs of failure.

**Figure 5:**
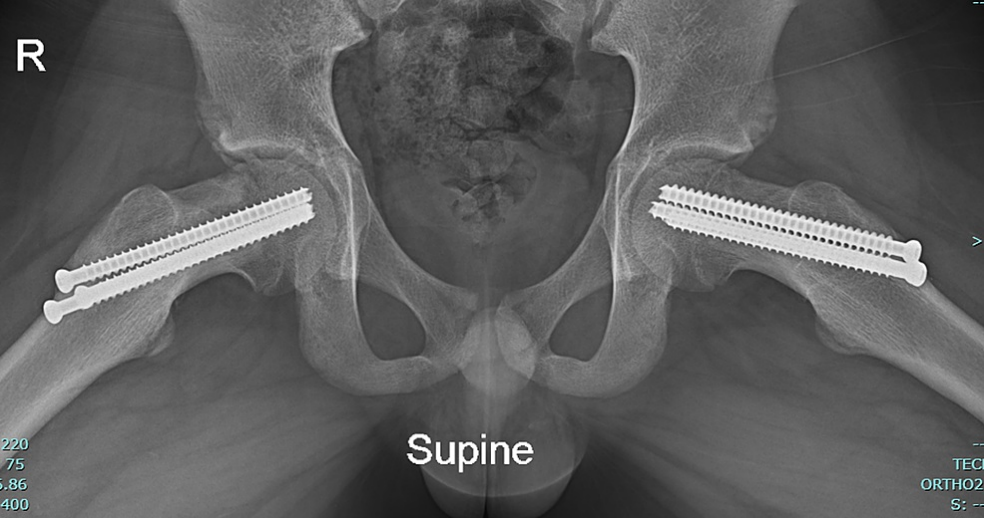
Bilateral frog leg radiographs at one year postoperatively.

At the 12-month follow-up, the patient was shown to be well-healed proximal femoral physis with a remodeled femoral neck on the left walking without pain (Figure 5).

## Discussion

Percutaneous placement of cannulated screws continues to be a mainstay for the treatment of SCFEs. However, the construct and placement of screws may need some rethinking. There have been risk factors regarding the progression of a slip without hardware failure [7], as well as multiple case reports of broken screws [5,6]. The failure of the screws, as seen in this case, is similar to the location seen in the other case reports. This location of hardware failure distal to the physis may occur due to the mobile segment of the epiphysis leading to continuous cyclical load the screw leading to failure in the cancellous bone of the femoral neck. The goal of screw placement is a complete epiphysiodesis of the proximal physis which was not achieved by the nine-month assessment images.

There are theoretical risks with multiple screw placements such as chondrolysis due to inadvertent joint penetration and prominence. However, multiple studies have also shown a biomechanical advantage to multiple screws [8]. It has been routinely accepted that multiple screws are appropriate for unstable SCFE. For stable fractures, one single centrally placed screw has presented the best risk-benefit profile. Ideally, a future study can help identify independent risk factors for failure helping a provider decide on treatment options. However, due to the low rate of occurrence, this is difficult to ascertain. Of the three cases cited in the literature, two were African American males. Two of the cases had issues with compliance with weight-bearing restrictions or follow-up. While this case is the only evidence of bilateral progression with a screw in place, one of the other three cases had unilateral progression.

If screw failure is noted early intervention is recommended. Even if screws initially only appear to be bent, the screws fracture on retrieval attempt, as occurred in our patient’s case. Prior case reports have suggested leaving the failed screw and supplementing with additional screws around the prior screw. We believe that extraction of the failed screw is best to allow easier placement of multiple new screws for greater stability. For cannulated screws, the conical extracting device is easy to place in the prior screw tract with fluoroscopic guidance. The reverse threads allow engagement of the distal screw segment and removal. When planning screw removal, our preferred technique is to provisionally pin the SCFE with an additional guidewire while removing the screw to prevent the possibility of further displacement due to the torque levels seen while using the conical extractor. If possible, using a wire that is compatible with the cannulated system of choice would allow for quick placement of a new screw after extraction of the failed screw. We opted for multiple screws at the second surgery for greater stability and to increase the chances of epiphysiodesis to occur at the proximal femoral physis. If revising a failed screw, the biomechanical advantage of multiple screws is preferable to just upsize a prior screw.

## Conclusions

Though rare, biomechanical failure of centrally placed screws in a mild stable SCFE is possible. Host factors and patient compliance may have an impact on failure, but there is a need for further investigation regarding the biomechanics of cannulated screw failure. If a failure occurs, it is our recommendation to extract the broken or bent screw and place multiple screws for stability and increased likelihood to lead to completion epiphysiodesis of the proximal femoral physis.
